# The regulation of the mitochondrial apoptotic pathway by glucocorticoid receptor in collaboration with Bcl-2 family proteins in developing T cells

**DOI:** 10.1007/s10495-016-1320-8

**Published:** 2016-11-26

**Authors:** Lilla Prenek, Ferenc Boldizsár, Réka Kugyelka, Emese Ugor, Gergely Berta, Péter Németh, Timea Berki

**Affiliations:** 1 Department of Immunology and Biotechnology, University of Pécs Medical Center, Szigeti út. 12., Pecs, 7624 Hungary; 2 Department of Medical Biology, University of Pécs Medical School, Szigeti út. 12., Pecs, 7624 Hungary

**Keywords:** Glucocorticoid receptor, Glucocorticoid hormone, Non-genomic pathway, Mitochondria, Thymocyte apoptosis, Bcl-2 proteins

## Abstract

Glucocorticoids (GC) are important in the regulation of selection and apoptosis of CD4^+^CD8^+^ double-positive (DP) thymocytes. The pronounced GC-sensitivity of DP thymocytes, observed earlier, might be due to the combination of classical (genomic) and alternative (non-genomic) glucocorticoid receptor (GR) signaling events modifying activation or apoptotic pathways. In particular, the previously demonstrated mitochondrial translocation of activated GR in DP thymocytes offered a fascinating explanation for their pronounced GC-induced apoptosis sensitivity. However, the fine molecular details how the mitochondrial translocation of GR might regulate apoptosis remained unclear. Therefore, in the present study, we intended to examine which apoptotic pathways could be involved in GC-induced thymocyte apoptosis. Furthermore we investigated the potential relationship between the GR and Bcl-2 proteins. Using an in vitro test system, thymocytes from 4-week-old *BALB*/*c* mice, were treated with the GC-analogue dexamethasone (DX). Bax accumulated in mitochondria upon DX treatment. Mitochondrial GR showed association with members of the Bcl-2 family: Bak, Bim, Bcl-x_L_. Elevated Cytochrome *C*, and active caspase-3, -8, and -9 levels were detected in thymocytes after DX treatment. These results support the hypothesis that in early phases of GC-induced thymocyte apoptosis, the mitochondrial pathway plays a crucial role, confirmed by the release of Cytochrome *C* and the activation of caspase-9. The activation of caspase-8 was presumably due to cross-talk between apoptotic signaling pathways. We propose that the GC-induced mitochondrial accumulation of Bax and the interaction between the GR and Bim, Bcl-x_L_ and Bak could play a role in the regulation of thymocyte apoptosis.

## Introduction

Despite their multiple side effects and broad organ-specificity, high-dose synthetic glucocorticoid hormone (GC) analogues are frequently used in the therapy of autoimmune diseases, hematological malignancies and allergies [1, 2]. GC analogues have been shown to promote apoptosis of leukemic cells and to trigger complex anti-inflammatory actions by targeting both the molecular and cellular components of the immune system [3, 4]. GCs induce apoptotic death of immature, developing thymocytes and also some groups of mature, activated T-cells [5]. In mouse models, GCs cause robust thymocyte depletion, primarily by the induction of CD4^+^CD8^+^ double positive (DP) thymocyte apoptosis [6–9].

Most of the GCs therapeutic actions are the results of their genomic effects mediated by the ligand-induced nuclear translocation of the cytoplasmic glucocorticoid receptors (GR) leading to the transactivation or -repression of numerous genes [10–13]. However, some effects, especially those at high GC concentrations, for example, used for intravenous pulse therapy or intraarticular injections, are too rapid to be mediated by changes at the genomic level which take hours or even days to develop. These “non-genomic”/alternative GC actions include the physicochemical interactions of the GC hormone with biological membranes [14] and the effects mediated by the glucocorticoid–glucocorticoid receptor (GC-GR) complex. These latter involves non-nuclear actions like rapid eNOS (endothelial nitrogen oxide synthase) activation or alterations in signaling events and effector mechanisms of the cells [15], for example the interaction of the activated GR with cytoplasmic proteins like NF-κB (nuclear factor-kappaB) [16], or with molecules of the TCR (T-cell receptor) signaling pathway like Lck (lymphocyte-specific protein tyrosine kinase), Fyn [17] and ZAP-70 (zeta-chain-associated protein kinase 70 kDa) [18]. The third non-genomic GC action is the translocation of GR to the mitochondria, which correlates to the sensitivity of a given cell type to GC-induced apoptosis [19, 20]. The GC-induced mitochondrial apoptotic pathway leads to the disruption of the mitochondrial membrane-potential and the release of key apoptosis inducing factors like Cytochrome *C* [21, 22]. This study focuses on this third type of accidental apoptotic cell death and its regulation.

The mitochondrial, or intrinsic, apoptotic pathway is regulated by pro-and anti-apoptotic members of the Bcl-2 protein family at the level of the mitochondria [23]. Within the pro-apoptotic members of the Bcl-2 family there are the Bcl-2 homology 3 (BH3)-only group proteins such as Bim, Bid, Bad, PUMA, Noxa, which transmit the apoptotic stimuli by activating Bax and Bak. The anti-apoptotic members such as Bcl-2 and Bcl-x_L_ counteract this process by binding and neutralizing the pro-apoptotic proteins. After Bax and Bak formed pores on the mitochondrial outer membrane, Cytochrome *C* is released and it participates in the formation of the apoptosome with Apaf1 and caspase-9 and activates caspase-3 [23, 24]. Caspase-3 can also be activated by caspase-8 after the initiation of the extrinsic apoptotic pathway [25, 26].

In a preliminary work, in a TCR transgenic mouse model, we have shown that thymocytes surviving during T cell selection up-regulated their mitochondrial anti-apoptotic Bcl-2 protein, suggesting that the mitochondria were directly involved in the regulation of thymocyte apoptosis [22]. Other studies with murine models have demonstrated the importance of Bax, Bak, Bim and Bcl-x_L_ in mediating dexamethasone (DX)-induced apoptosis [27, 28]. Previously we have shown, that upon short-term in vitro exposure of DP thymocytes to GCs the GR translocated to the mitochondria within 30 min, having a direct effect on the mitochondrial function and decreasing the mitochondrial membrane potential [6]. Taking these preliminary data together, we hypothesize that the mitochondrial GR translocation could play an important role in the GC-induced apoptosis of thymocytes. On the other hand the relation of Bcl-2 family proteins like Bak, Bax, Bim or Bcl-x_L_ with the GR has not been investigated so far in the GC-induced mitochondrial apoptotic pathway of thymocytes.

Therefore, in this study, we analyzed the short term in vitro DX treatment-induced interactions between the GR and Bcl-2 family member proteins in mouse thymocytes, paying special attention to their distribution between the cytoplasm and mitochondria. Parallel with this we characterized the activation of different caspases as markers of apoptosis. Here, we provide evidence for the activation of the mitochondrial apoptotic pathway as well as direct association between the GR and Bak, Bim, and Bcl-x_L_ after short term GC analogue treatment in thymocytes.

## Materials and methods

### Mice

3–4 weeks old *BALB*/*c* mice (obtained from The Jackson Laboratory, Bar Harbor, ME, USA) were kept under conventional conditions and provided with pelleted rodent chow and water ad libitum. All animal experiments were carried out in accordance with the regulations of Committee on Animal Experimentations of University of Pécs (#BA 02/2000–16/2015).

### Short-term in vitro GC-analogue treatment of isolated thymocytes

After sacrifice, thymi were removed and homogenized mechanically in RPMI-1640 medium (Sigma-Aldrich, Budapest, Hungary) followed by filtration through nylon mesh. Cell viability was determined by trypan-blue dye exclusion test using a hemocytometer. 5 × 10^7^ thymocytes were treated with 10^−6^ M DX (synthetic steroid compound, which has primarily GC-like effects), 10^−2^ M stock dissolved in dimethyl sulfoxide [(DMSO), both from Sigma-Aldrich] in serum-free RPMI for 1 and 3 h for western blotting, 0.5 h for confocal microscopy and for 0.5, 1, 2 and 3 h for flow cytometry at 37 °C. Control samples were kept under the same conditions for the same time in the presence of the solvent alone. The treatment was stopped by adding ice-cold phosphate buffered saline (PBS), containing 0.1 % NaN3 (Sigma-Aldrich).

### Antibodies

The following antibodies (Abs) were used for flow cytometry: anti-CD4-Phycoerythrin-Cyanine5 (PE-Cy5) (clone# RM4–5) and anti-CD8-Phycoerythrin (PE) (clone# 53–6.7) (all from BD Pharmingen, San Jose, CA, USA), for analysis of activated (cleaved) caspases rabbit anti-caspase-3 (clone# 5A1E), rabbit anti-caspase-8 (clone# D5B2) and rabbit anti-caspase-9 (all from Cell Signaling Technology, Danvers, MA, USA) were used with anti-rabbit IgG-Fluorescein (FITC) (Sigma-Aldrich) as secondary Ab.

For confocal microscopy the following Abs were used: anti-CD4-Pacific Blue (clone# RM4–5, BD Pharmingen), anti-CD8-Pacific Orange (clone# 5H10, Life Technologies, Waltham, MA, USA), anti-GR-FITC (clone# 5E4-B1, produced in our laboratory) [29] and rabbit anti-Bak, -Bax, -Bcl-x_L_ (all from Santa Cruz Biotechnology, Dallas, TX, USA) and -Bim (clone# C34C5, Cell Signaling Technology) with goat anti-rabbit IgG-Cyanine3 (Cy3) secondary Ab and goat anti-rabbit IgG-FITC secondary Ab (Sigma-Aldrich).

For western blot analysis of the activated (cleaved) caspases in the subcellular fractions the following Abs were used: rabbit anti-caspase-3, -8, and -9 (all from Cell Signaling Technology) in 1:1000 dilutions. The pro-apoptotic proteins were detected with mouse anti-Cytochrome *C* (clone# 7H8.2C12, BD Pharmingen) in 1:2000 dilution, rabbit anti-Bax (Santa Cruz Biotechnology) in 1:500 dilution. For reprobing mouse anti-β-actin (clone# AC-74, Sigma-Aldrich) in 1:5000 dilution and anti-Cytochrome *C* Abs were used.

For immunoprecipitation, anti-GR (clone# 8E9, produced in our laboratory) [29] was used. For western-blot analysis of immunoprecipitated samples the following primary Abs were used: anti-Bak, anti-Bax (both from Santa Cruz Biotechnology), mouse anti-Bcl-x_L_ (BD Pharmingen) in 1:1000 dilution, rabbit anti-Bim (Cell Signaling Technology) in 1:1000 dilution and mouse anti-GR (clone# 5E4, produced in our laboratory) in 1:2000 dilution [29].

For visualization of the western blots peroxidase conjugated anti-mouse- or anti-rabbit IgG (produced in our laboratory) were used as secondary Abs in 1:1000 dilutions.

### Subcellular fractionation

Mitochondria Isolation Kit (Pierce, Rockford, IL, USA) was used to separate cytoplasmic, mitochondrial and nuclear fraction from thymocytes, according to manufacturer’s instructions, with minor modifications according to Stasik et al. [30]. Briefly, isolated solvent control and DX-treated thymocytes were washed in cold PBS-azide (PBS containing 0.1 % NaN_3_) and lysed. After centrifugation at 800×*g* for 10 min, the nuclear pellet was separated. The post-nuclear supernatant was centrifuged first at 3000×*g* for 15 min and then at 12,000×*g* for 5 min. The pellet containing mitochondria was either dissolved in sodium dodecyl sulfate (SDS) sample buffer (125 mM Tris, 4 % SDS, 10 % mercaptoethanol, 0.006 % bromo-phenol-blue (all from Sigma-Aldrich) and 10 % glycerol (Molar Chemicals, Budapest, Hungary)) or used for immunoprecipitation and the clear supernatant was used as a cytosolic fraction. The supernatant was either used for immunoprecipitation or boiled immediately in SDS sample buffer for 10 min. To use mitochondria for immunoprecipitation the pellet was lysed in TEGM lysis buffer (10 mM Tris base, 4 mM EDTA (all from Sigma-Aldrich), 50 mM sodium chloride, 20 mM sodium molibdate (Molar Chemicals), 10 % glycerol, pH 7.6) complemented freshly with protease inhibitor and Na-orthovanadate (both from Sigma-Aldrich). The samples were frozen and thawed five times in liquid nitrogen and then incubated for 30 min on ice and centrifuged for 10 min at 13,000 rpm and the supernatant was used for immunoprecipitation.

### Immunoprecipitation

For immunoprecipitation, the cytosolic and mitochondrial fractions were incubated overnight under continuous rotation with the appropriate amount of precipitating antibodies (see in “Antibodies” section) in blocking buffer (10 mM Tris, 100 mM sodium chloride, pH 7.4 containing 10 % bovine serum albumin (BSA, Sigma-Aldrich)); then Protein-G (Santa Cruz Biotechnology) was added to the samples and they were incubated for additional 2 h under continuous rotation. Finally, samples were washed five times in PBS and immune complexes were removed from the Protein-G with boiling for 3 min in SDS sample buffer.

### Western blotting

Cell fractions were subjected to sodium dodecyl sulfate polyacrylamide gel electrophoresis (SDS-PAGE) on a 10 or 15 % gel. The gels were blotted for 2 h to nitrocellulose membranes using Mini Trans-Blot Cell blotting equipment (both from Bio-Rad, Hercules, CA, USA). After transfer, nitrocellulose membranes were soaked in blocking buffer (2 % BSA or 1 % non-fat dry milk (Bio-Rad), 10 mM Tris, 100 mM sodium chloride and 0.1 % Tween 20 (Molar Chemicals), pH 7.4) and then incubated with the appropriate primary antibodies. Anti-β-actin and anti-Cytochrome *C* antibodies were used to control the equal loading and purity of the fractions. Blots were then probed with the appropriate secondary Abs. Blots were washed in a buffer containing 10 mM Tris, 100 mM sodium chloride and 0.1 % Tween 20 (pH 7.4). Western blot visualization was performed by enhanced chemiluminescence as described in the manufacturer’s instructions (SuperSignal West Femto Chemiluminescent substrate, Pierce). Luminescent light signals were detected with Fujifilm LAS 4000 blot documentary system.

### Analysis of blots

Densitometry of blots was done with the Image J software (http://rsb.info.nih.gov/ij). Densitometric data was calculated using the original, unmodified images. Relative densities of caspases and cytoplasmic Bax blots were normalized to the relative densities of β-actin, the mitochondrial fraction of Bax to Cytochrome *C* to determine the relative expression in the subcellular fractions. Relative densities of Bim, Bak, and Bcl-x_L_ immunoprecipitation blots were normalized to the relative densities of GR. Brightness and contrast of representative images have been adjusted.

### Labeling cells for confocal laser scanning microscopy

After 30 min DX treatment CD4-Pacific Blue and CD8-Pacific Orange labeling of thymocytes was performed in binding buffer (PBS containing 0.1 % BSA and 0.1 % NaN_3_) then cells were fixed in 4 % paraformaldehyde (Sigma-Aldrich) and washed in permeabilization buffer (PBS containing 0.1 % BSA, 0.1 % NaN_3_ and 0.1 % saponin (Sigma-Aldrich)). The intracellular labeling of the cells was performed in saponin buffer with rabbit anti-Bak, Bax, Bcl-x_L,_ and Bim as primary Abs and anti-rabbit IgG-Cy3 as secondary Ab then with 1 µg/ml anti-GR-FITC antibody [29]. The cells were incubated for 1 h with the Abs and washed twice in saponin buffer. After the labelling the cells were washed again twice in saponin buffer and once with PBS then cytospined onto slides. The excess fluid was carefully aspirated and the slides were covered using Promofluor Antifade Reagent (PromoKine, Heidelberg, Germany).

### Mitotracker chloromethyl-X-rosamine (CMX-Ros) staining of mitochondria for confocal laser scanning microscopy

CMX-Ros (Invitrogen, Waltham, MA, USA) is a cell-permeant lipophilic reagent, which diffuses through the plasma membrane and accumulates in active mitochondria due to normal mitochondrial membrane potential [31]. Briefly, 10^6^ thymocytes were incubated in 1 ml serum-free RPMI containing 10 µl CMX-Ros stock solution (1 µg/ml in DMSO) for 30 min at 37 °C, following the manufacturer’s instructions, parallel with 1 µM DX treatment. Cell surface labelling with anti-CD4-Pacific Blue and anti-CD8-Pacific Orange and intracellular labeling with rabbit anti-Bax as primary Ab, and anti-rabbit IgG-FITC as secondary Ab was performed as indicated in “Labeling cells for confocal laser scanning microscopy” section.

### Confocal microscopic image acquisition and analysis

Visualization and analysis of the samples were carried out using an Olympus Fluoview 300 confocal microscope with an Olympus Fluoview FV1000S-IX81 image acquisition software system. Data were collected in four separate channels, including differential interference contrast (DIC), UV for CD4, virtual red for CD8, FITC for GR, red for Bak, Bax, Bcl-x_L,_ and Bim or red for mitochondria and FITC for Bax. Sequential scanning was used for image acquisition. Signals were collected from cells in 3–3 frames and Bak, Bax, Bcl-x_L,_ Bim-GR and CMX-Ros-Bax morphological association was analyzed with the ImageJ software (http://rsb.info.nih.gov/ij) using co-localization plug-in. Co-localization data was calculated using the original, unmodified images. Based on the analysis of pixel fluorescence intensities, ranging from 0 to 255, specific staining was distinguished from background by using a threshold value of 50 as described elsewhere [32, 33]. Then, co-localized pixels between Cy3-GR and CMX-Ros-Bax were counted. One hundred DP cells per sample were analyzed altogether using this approach. Brightness and contrast of representative images have been adjusted.

### Labeling cells for flow cytometry

10^6^ cells were treated with DX for 0.5, 1, 2 and 3 h. Cell surface labelling with CD4-PECy5 and CD8-PE and intracellular labelling with rabbit anti-caspase-3, -8, -9 as primary antibodies and with anti-rabbit IgG-FITC as secondary antibody was performed as indicated in “Labeling cells for confocal laser scanning microscopy” section followed by flow cytometric analysis.

### Flow cytometric data acquisition and analysis

Samples were measured and analyzed in a FACSCalibur flow cytometer (Becton Dickinson, San Jose, CA, USA), using the CellQuest Pro software. Thymocyte subpopulations were analyzed separately based on their cell surface CD4/CD8 expression for FITC intensity detected in the FL1 channel. Fluorescent histogram plots were used to compare the ratio of active caspase-3, -8, -9 expressing cells (FITC positive) of different samples.

### Statistical analysis

Data are presented as mean ± SEM. GraphPad Prism (version 6.01, GraphPad Software, La Jolla, CA) program was used to create the artwork and perform the statistical analysis using Student’s *t* test. *p* < 0.05 was considered statistically significant.

## Results

### DX-induced changes in the co-localization between GR and members of Bcl-2 protein family

**Fig. 1:**
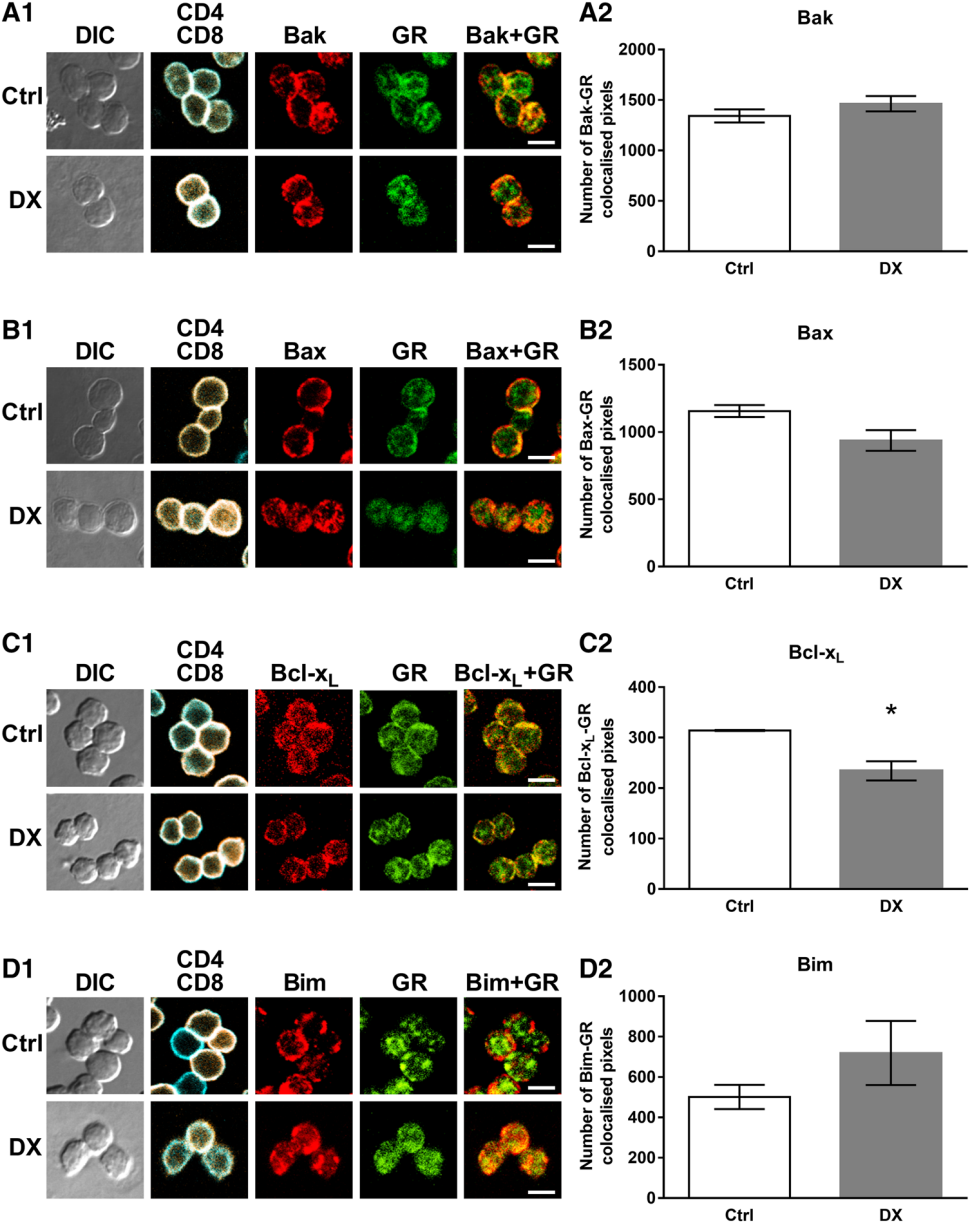
Co-localization of the GR with members of the Bcl-2 protein family: Bak, Bax, Bcl-x_L_ and Bim in DP thymocytes. Representative confocal microscopic images from at least three independent experiments showing GR-Bak (A1), GR-Bax (B1), GR-Bcl-x_L_ (C1) and GR-Bim (D1) co-localization in control (Ctrl) and 30 min DX-treated cells. DIC, CD4 (*blue* channel) and CD8 (virtual *red* channel) overlaid, intracellular GR (*green* channel) and Bak, Bax, Bcl-x_L_, Bim (*red* channel) images are shown. The co-localization of the GR with Bak, Bax, Bcl-x_L_ and Bim (GR-Bak, Bax, Bcl-x_L_, Bim merged images) is indicated by the *yellow areas. Scale bars* 8 µm each. *Bar diagrams* show the quantification of the changes in the GR-Bak (A2), GR-Bax (B2), GR-Bcl-x_L_ (C2) and GR-Bim (D2) co-localization in DP thymocytes after in vitro DX treatment. *Bars* represent the number of co-localized pixels per cell as calculated by the co-localization plugin of the ImageJ software. The mean ± SEM was calculated from the data of 100 DP cells per treatment, respectively. Significant changes (*p* < 0.05) in DX-treated cells versus controls are indicated by *asterisk*. (Color figure online)

Previous studies have shown the importance of Bax, Bak and Bim in mediating DX-induced apoptosis [27, 28, 34–36] and in our preliminary work, in a TCR transgenic model, we have observed that thymocytes, surviving T cell selection, up-regulated their Bcl-2 protein level [22]. We have also shown, that upon in vitro exposure of DP thymocytes to GC the activated GR translocated to the mitochondria within 30 min which was followed by the decrease of the mitochondrial membrane potential [6], indicating the importance of non-genomic effects and the mitochondrial apoptotic pathway in the GC-induced apoptosis of thymocytes. Therefore, now we set out to find potential molecular partners for the activated GR in the mitochondrial apoptotic pathway. To this end we investigated possible protein interactions between the GR and Bcl-2 family proteins, which are responsible for the control of the mitochondrial membrane potential [37]. To test our hypothesis the co-localization of GR and Bak, Bax, Bcl-x_L_ or Bim was analyzed in DP thymocytes before and after 30 min of high dose DX treatment (Fig. 1). We found that the GR co-localized to some extent with all four investigated Bcl-2 family proteins (Fig. 1a1–d1). Upon DX treatment the GR-Bak association showed minimal change (Fig. 1a1), the GR-Bax, -Bcl-x_L_ association decreased (Fig. 1b1, c1), while the GR-Bim association increased (Fig. 1d1).

To quantify the rate of co-localization, we calculated and compared the number of co-localized pixels in individual DP cells after 30 min of DX treatment to their controls. After DX treatment the co-localized pixel number minimally changed between Bak and GR (1463 ± 76 versus 1342 ± 65 in the control) (Fig. 1a2) but decreased slightly between Bax and GR (937 ± 77 versus 1156 ± 44 in the control) (Fig. 1b2). The co-localization between GR and Bcl-x_L_ decreased significantly after DX treatment (234 ± 19 versus 314 ± 1 in the control) (Fig. 1c2). We observed a remarkable, but statistically not significant, increase in the co-localization of Bim and GR upon 30 min DX treatment (719 ± 159 versus 501 ± 60 in the control) (Fig. 1d2).

### The GR interacts with members of the Bcl-2 protein family in the cytoplasm and the mitochondria of thymocytes

**Fig. 2:**
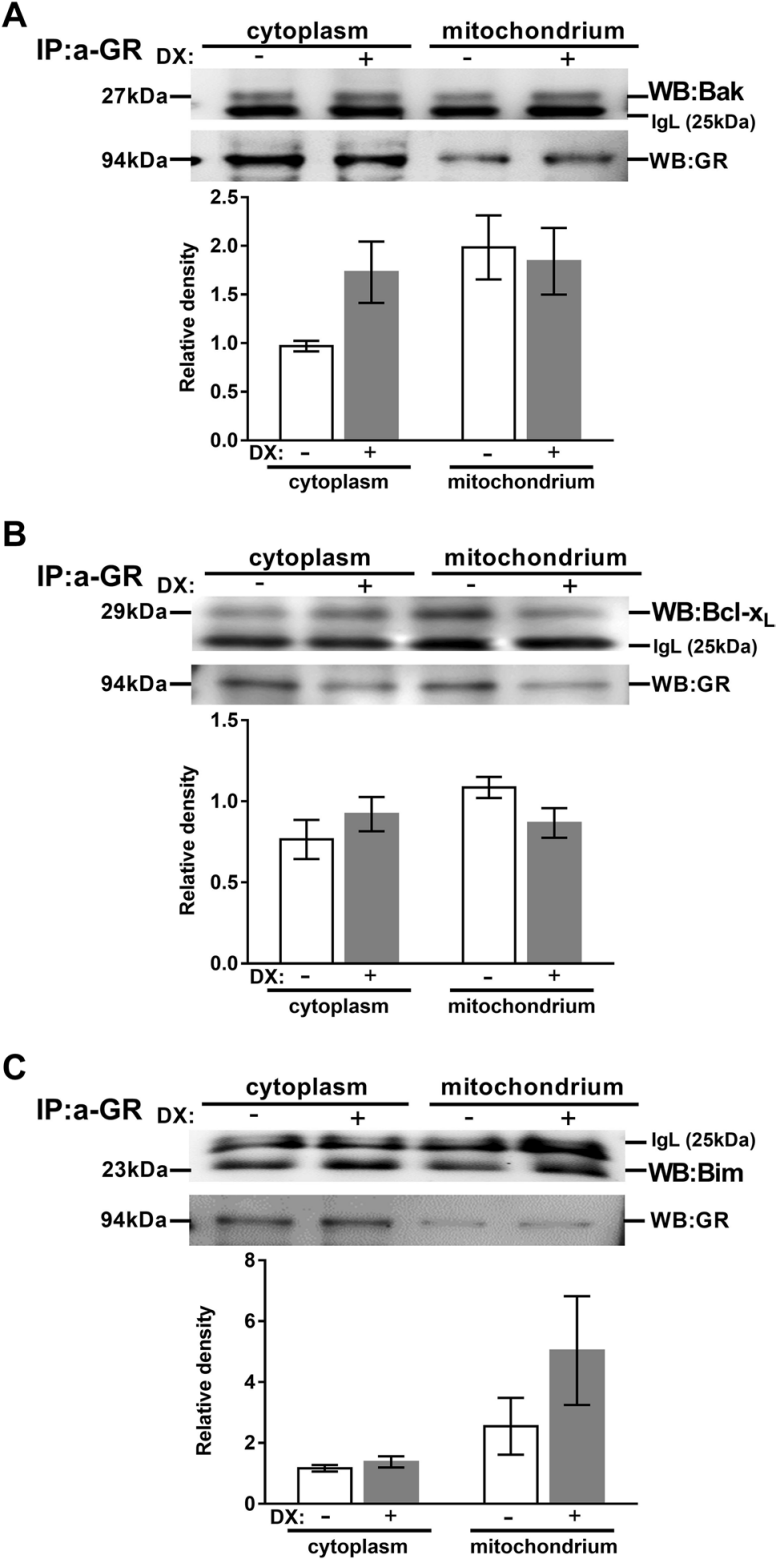
Association of the GR with members of the Bcl-2 family in thymocytes. Anti-Bak (**a**), Bcl-x_L_ (**b**) and Bim (**c**) western blots are shown from cytoplasmic and mitochondrial fractions of thymocyte lysates after anti-GR precipitation with or without DX treatement. Blots were reprobed with anti-GR antibody to confirm equal loading of the samples. The figure shows representative blots and densitometry data of at least three independent experiments. *Diagrams below each blot* show the relative Bak, Bcl-x_L_ and Bim levels in the cytoplasm (normalized to GR) and the mitochondria (normalized to GR). *Bars* represent the mean ± SEM of relative densities compared with the controls. IgL: immunoglobulin light chain

To confirm our confocal microscopic results, we investigated the interaction of the GR with Bcl-2 family member proteins: Bak, Bax, Bcl-x_L_ and Bim proteins in thymocytes using co-immunoprecipitation with anti-GR antibody. We also wanted to elucidate whether the high dose DX treatment changed the active GR-Bcl-2 family protein complexes’ subcellular distribution. Therefore, we performed subcellular fractionation and isolated cytoplasmic and mitochondrial fractions from 30 min DX or vehicle-treated, unseparated thymocytes. After subcellular fractionation immunoprecipitation was performed with anti-GR antibody and then the samples were further analyzed by western blot to visualize the co-precipitated Bcl-2 family proteins. Densitometric quantification of western blots was carried out. The Bcl-2 family protein levels were compared in both untreated and DX-treated samples. Note: although thymocytes were not separated, based on their cell surface phenotype, in these experiments, 70–80 % of the cells are DP in 3-to-4-week-old *BALB*/*c* mice [38]; therefore the results from our immunoprecipitation and western blot experiments give a good impression about the DP cells. Results of representative experiments are shown in Fig. 2. Confirming our confocal microscopic data (see “DX-induced changes in the co-localization between GR and members of Bcl-2 protein family” section), association of the GR with Bak, Bim, Bcl-x_L_ proteins could be observed both in the cytoplasmic and mitochondrial fractions of both untreated and DX-treated thymocytes (Fig. 2a–c, respectively), however, Bax protein did not show any direct association with the GR (data not shown).

Bak co-precipitated with the GR, and upon DX treatment the Bak-GR co-precipitation increased in the cytoplasmic and slightly changed in the mitochondrial fraction (Fig. 2a). We also observed the co-precipitation of Bim with the GR (Fig. 2b). Bcl-x_L_ also co-precipitated with GR (Fig. 2b). The rate of their co-precipitation increased in the cytoplasmic and decreased in the mitochondrial fraction upon DX treatment in comparison to the control (Fig. 2b). Finally, the GR-Bim association changed only minimally in the cytoplasmic fraction, but remarkably increased in the mitochondrial compartment (Fig. 2c). This pronounced mitochondrial accumulation of Bim suggests its potential role in the mitochondrial (intrinsic) apoptotic pathway in the GC-induced thymocyte apoptosis.

### DX treatment-induced mitochondrial accumulation of Bax

**Fig. 3:**
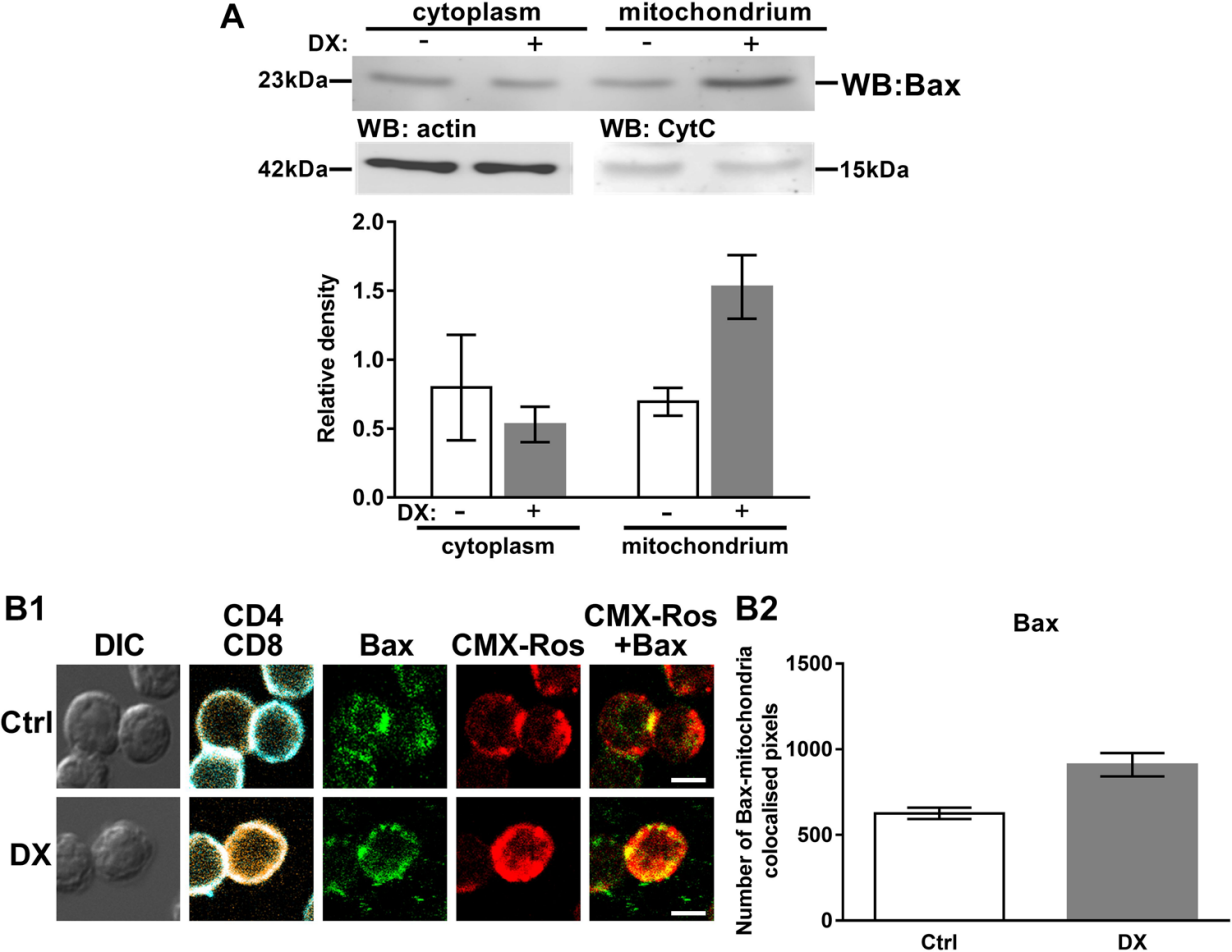
Subcellular distribution of Bax in thymocytes upon DX treatment. **a** Western blot shows the DX treatment-induced redistribution of Bax between the cytoplasmic and mitochondrial fractions of thymocytes. Blots were reprobed with anti-β-actin or anti-Cytochrome *C* (Cyt *C*) antibodies to confirm the purity of the cytoplasmic and mitochondrial fractions, respectively. The *figure* shows a representative blot and the densitometry data of at least three independent experiments. The *diagram* shows the relative Bax expression in the cytoplasm (normalized to β-actin) and the mitochondria (normalized to Cytochrome *C*). *Bars* represent the mean ± SEM of relative densities compared to the controls. **b** Mitochondrial translocation of Bax in DP thymocytes. **b**1 Representative confocal microscopic images of at least three independent experiment showing CMX-Ros-Bax co-localization in control (Ctrl) and 30 min DX-treated cells. DIC, CD4 (*blue* channel) and CD8 (virtual *red* channel) overlaid, mitochondria (CMX-Ros, *red* channel) and Bax (*green* channel) images are shown. The co-localization of mitochondria with Bax (CMX-Ros-Bax merged images) is indicated by *yellow areas. Scale bars* are 8 µm each. **b**2 Quantification of the changes in the CMX-Ros-Bax co-localization in DP thymocytes after in vitro DX treatment was performed using the co-localization plugin of the ImageJ software. *Bars* represent the number of co-localized pixels. The mean ± SEM was calculated from the data of 100 DP cells per treatment, respectively. (Color figure online)

Bax is a key pro-apoptotic protein in the mitochondrial apoptotic pathway. It has been shown earlier, that Bax has a constant turnover between the mitochondrial membrane and the cytoplasm [39] and it has also been demonstrated to be important in GC-induced apoptosis together with Bak [27, 28, 34]. In the case of Bax we could not confirm the co-localization, observed by confocal microscopy, with co-immunoprecipitation (data not shown). Therefore, we investigated whether the high dose DX treatment caused any redistribution of Bax between the cytoplasmic and mitochondrial fractions of thymocytes, and we have found that Bax accumulated in the mitochondrial fraction after 30 min of DX treatment, compared to the control (Fig. 3a). This result was confirmed by confocal microscopy (Fig. 3b1); the number of Bax-CMX-Ros co-localized pixel number increased upon 30 min of DX treatment (910 ± 68 versus 626 ± 33 in the control) (Fig. 3b2).

### Kinetics of caspases’ activation in DP thymocytes

Preceding studies with knock-out (KO) models have shown the importance of the intrinsic apoptotic pathway in GC-induced apoptosis of thymocytes [40, 41]. However, others have emphasized the role of caspase-8 and the extrinsic pathway in this process [42, 43]. In our previous research, with DP thymocytes, we have shown that the translocation of GR to the mitochondria was followed by the decrease of the mitochondrial membrane potential [6], which supported the significance of the mitochondrial apoptotic pathway in DP thymocyte apoptosis induced by GCs.

**Fig. 4:**
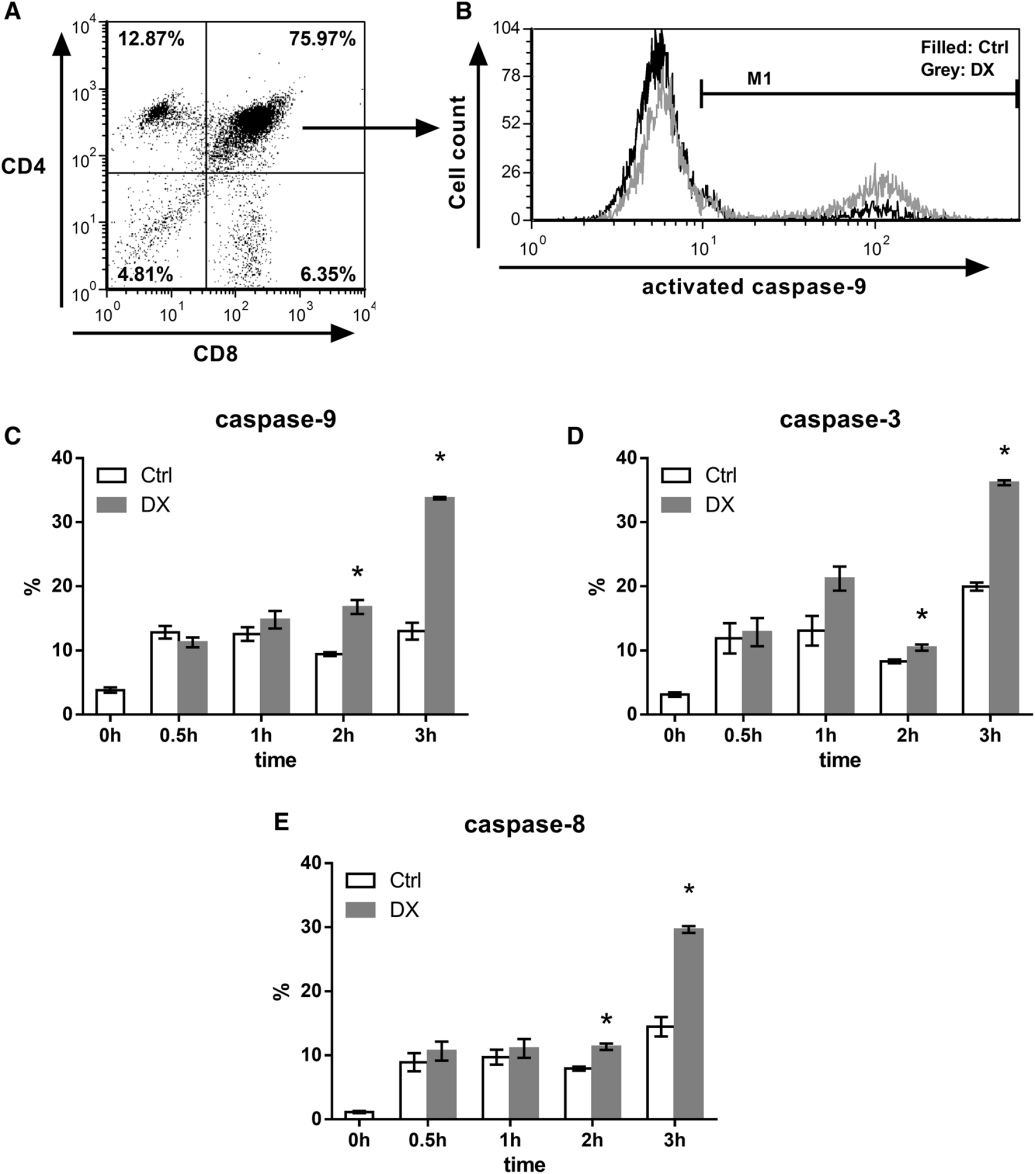
Flow cytometric analysis of the kinetics of caspase activation in DP thymocytes upon 30 min to 3 h of DX treatment. **a** Thymocyte subpopulations were gated based on their CD4/CD8 expression. **b** The representative fluorescent histogram plot shows the active caspase-9 positive percentage of DP thymocyte subpopulation before and after 3 h of DX treatment. *Bar diagrams* show the mean ± SEM of cleaved, active caspase-9 (**c**), -3 (**d**) and -8 (**e**) positive percentage of cells (calculated from the data of three animals) in the DP thymocyte population and its changes upon 30 min to 3 h of DX treatment. Significant (*p* < 0.05) differences compared to the untreated controls are indicated by *asterisk*

Hence, now to investigate the activation of caspases in DP thymocytes, separately from other thymocyte subpopulations, we examined the activation of caspase-3,-8, and -9 after 0.5, 1, 2 and 3 h of DX treatment in DP thymocytes (Fig. 4). The ratio of DP cells containing cleaved caspase-9 increased significantly after 2 and 3 h of DX treatment (Fig. 4c). The percentage of DP cells in which active caspase-3 was detected showed increase already after 1 h of DX treatment, and after 2 and 3 h of DX treatment the rate of DP cells having active caspase-3 increased significantly (Fig. 4d). The activation of caspase-9 together with the cleavage of caspase-3 implied the activation of the intrinsic, mitochondrial apoptotic pathway upon DX treatment. The ratio of active caspase-8 containing DP cells was slightly elevated upon 0.5 and 1 h of DX treatment, and this increase continued and became significant after 2 and 3 h DX treatment showing a similar tendency to the active caspase-9 (Fig. 4e). The changes in caspase-9 activation seemed to be more pronounced than in the case of caspase-8 after 2 and 3 h of DX treatment, which may suggest a pivotal role of caspase-9 in DX-induced thymocyte apoptosis.

### DX-induced caspase activation and Cytochrome *C* release to the cytoplasm in thymocytes

**Fig. 5:**
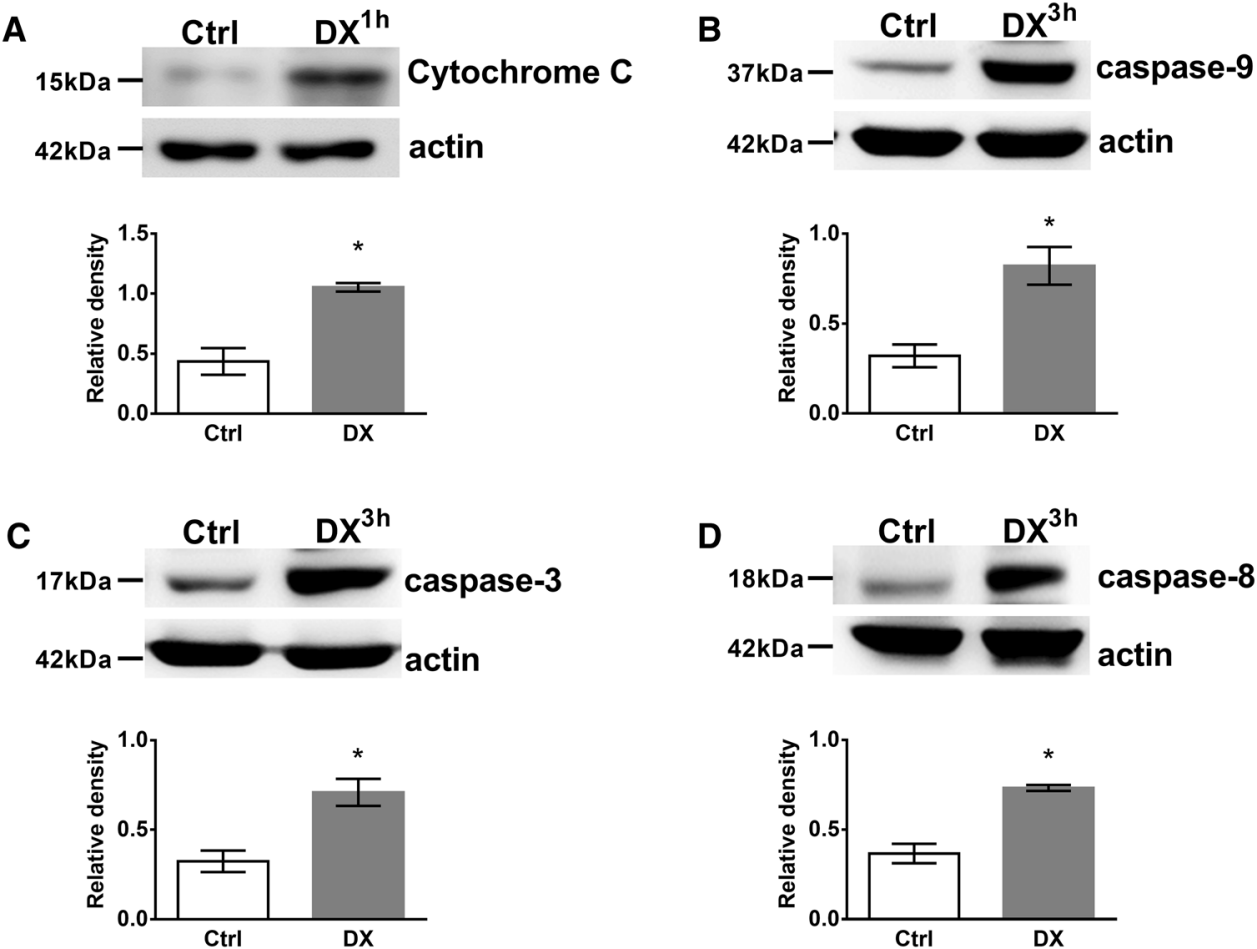
Western blot analysis of DX treatment induced Cytochrome *C* release to the cytoplasm and caspase-3, -8, -9 activation in thymocytes. The cytoplasmic presence of Cytochrome *C* (**a**), active (cleaved)-caspase-9 (**b**), -3 (**c**) and -8 (**d**) were detected in thymocyte lysates by western blot. Blots were reprobed with anti-β-actin antibody to confirm equal loading of the samples. The *figure* shows representative blots and the densitometry data of at least three independent experiments. *Diagrams below each blot* show the relative Cytochrome *C* and caspase-9, -3, -8 levels (normalized to β-actin). *Bars* represent the mean ± SEM of relative densities compared to the untreated controls. Significant (*p* < 0.05) differences are indicated by *asterisk*

To confirm our flow cytometric results we performed western blot analysis of activated caspases in unseparated thymocytes (70–80 % of the cells are DP [38]) upon 3 h of DX treatment (which was the peak activation seen with flow cytometry, see Fig. 4) together with the analysis of Cytochrome *C* release to the cytoplasm after 1 h DX treatment. Cell lysates of untreated, control, and in vitro DX-treated thymocytes were compared for active caspase-3, -8, -9 and Cytochrome *C* levels (Fig. 5). 1 h, high dose DX treatment caused the significant increase of Cytochrome *C* level in the cytoplasm (Fig. 5a). We observed the significant elevation of active caspase-9, -3 levels (Fig. 5b, c, respectively) compared to the control after 3 h of high dose DX treatment which are characteristic signs of the activation of the intrinsic (mitochondrial) apoptotic pathway. Interestingly, the initiator caspase-8 of the extrinsic pathway was also significantly elevated upon DX treatment (Fig. 5d), which might reflect a cross-talk between the intrinsic- and extrinsic pathways or may indicate the activation of another parallel apoptotic pathway.

## Discussion

Glucocorticoid receptor (GR) signaling plays an important regulatory role in the selection and apoptosis of thymocytes [6–8]. Besides the nuclear-, mitochondrial translocation of the ligand-bound GR might dictate GC-induced apoptosis sensitivity of the cells [6, 44–49]. In a previous study, we followed the ligand-induced GR trafficking in GC-sensitive CD4^+^CD8^+^ DP thymocytes [50–52] upon short term in vitro GC treatment and demonstrated the GR translocation into the mitochondria, which correlated well with their pronounced GC-induced apoptosis sensitivity [6, 51]. However, the molecular events following the short-term GC treatment-induced mitochondrial translocation remained to be elucidated. In our present work we clarified that the GR regulates the mitochondrial apoptotic pathway of thymocytes in close collaboration with the Bcl-2 family proteins.

We observed both co-localization and direct molecular association of Bak with GR (Figs. 1, 2). After DX treatment this association was unchanged in the mitochondrial fraction but increased in the cytoplasm of thymocytes upon high-dose short-term DX treatment. Upon apoptotic stimuli, Bax translocates to the mitochondria where it forms a complex with Bak leading to mitochondrial pore formation [24]. Our findings suggest that Bax has a primary role in the early phase of DX-induced apoptosis of thymocytes, although not associating directly with the GR. We cannot rule out the possibility that Bak also plays a role in GC-induced apoptosis, but probably joins at a later stage than we examined in our work. This is supported by earlier observations in thymocytes form Bax/Bak double KO mice which were completely resistant to GC-induced apoptosis, whereas Bax or Bak single KO mice thymocytes were still sensitive to GCs [34]. These studies, with knock-out mice, have strengthened the importance of Bak in GC-induced apoptosis, but also have suggested that Bax and Bak may compensate for each other [34].

Our results showed the association of Bim, a BH3-only protein, with GR and their interaction increased especially in the mitochondrial fraction upon DX treatment (Figs. 1, 2). Bim^−/−^ knock-out mice showed impaired GC-induced apoptosis [35, 36] showing its important but not exclusive participation in this death process. This is also supported by the results of other research groups [53]. GCs have been found to induce the expression of Bim in murine thymocytes after 2 or 3 h of DX treatment [47, 54]. Increased expression of Bim has correlated with increased sensitivity to GC-induced apoptosis [55, 56], dysregulation of its gene expression has been found in solid and hematopoietic malignances [57], where reduced expression correlated with increased disease risk [58], and single nucleotide polymorphisms have been associated with impaired responsiveness to anticancer therapies [59–61]. Our results also support that Bim plays a crucial role in the initiation of GC-induced apoptosis of DP thymocytes; the increased association of Bim with the GR in the mitochondria may promote the activation and oligomerization of Bax in the mitochondrial outer membrane.

Interestingly, we also observed interaction between Bcl-x_L_, an anti-apoptotic member of the Bcl-2 family, and the GR during the DX-induced apoptotic processes. Bcl-x_L_ has been shown to retrotranslocate Bax from the mitochondria to the cytoplasm by binding to it and thus inhibiting its pro-apoptotic activity [62]. We hypothesize that the interaction between the GR and Bcl-x_L_ would cause the inhibition of this particular Bcl-x_L_ function. After 30 min DX treatment the GR bound ratio of Bcl-x_L_ increased in the cytoplasmic but decreased in the mitochondrial fraction (Fig. 2) which suggests that Bcl-x_L_, after translocating to the cytoplasm from the mitochondria, binds to the GR, and this sequestration could abolish its antagonistic effect on the apoptotic process. This hypothesis about the inhibitory effect of the GR on Bcl-x_L_ is supported by the result of another research group where it has been observed that the expression of Bcl-x_L_ decreased significantly after 2 or 3 h of DX treatment [54]. However, the co-localization between Bcl-x_L_ and the GR decreased significantly after DX treatment (Fig. 1), which might be due to the fact that the co-localization results are only from DP cells and it gives the overall ratio of co-localization, both in the cytoplasm and the mitochondria, while unseparated thymocytes were used for the co-immunoprecipitation experiment and the cytoplasmic and mitochondrial fractions were analyzed separately.

The rate of co-localization between Bax and the GR slightly changed upon DX treatment (Fig. 1), but we could not confirm the co-localization, observed by confocal microscopy, with co-immunoprecipitation experiments. Co-localization expresses molecular proximity, but does not reflect necessarily direct molecular interaction between two molecules. In the case of Bax, where the co-localization with the GR was not confirmed by co-immunoprecipitation, the results suggest that the two molecules were very close to each other, but there were no direct interaction between them. According to our results GR, a 94 kDa molecule, associates with other members of the Bcl-2 protein family, which are in the vicinity of Bax. It is known from the work of others [62–65] that these Bcl-2 proteins interact with each other, which may explain the proximity of the GR to Bax without direct association. Besides we detected a clear redistribution of Bax from the cytoplasm to the mitochondria (Fig. 3) which correlated with the results of others [27, 28] suggesting the central role of Bax in DX-induced apoptosis of thymocytes. Bax trafficking between the mitochondrial outer membrane and the cytoplasm is a key regulator of the intrinsic (mitochondrial) pathway of apoptosis [24, 39, 66]. Bax oligomerization in the mitochondrial membrane leads to the formation of a permeability pore, which causes the decrease of the mitochondrial membrane potential [24], as it has been detected in our previous experiments [6].

Caspases are important effectors of both, intrinsic and extrinsic, apoptotic pathways [23, 26]. In our experiments we analyzed the kinetics of caspases’ activation from 0.5 to 3 h of DX treatment. We observed significantly increased number of DP thymocytes containing active, cleaved caspase-3, -8, -9 after 2 and 3 h of DX treatment. After 1 h of DX treatment, the caspase-3 activation was probably the result of caspase-9 activation following the decrease of the mitochondrial membrane potential observed after 30 min DX treatment in our previous work [6]. But the activation of caspase-3 after 1 h of DX treatment may be partially the result of the activation of parallel apoptotic pathways. These include ceramide and sphingosine generation which were reported to be able to induce caspase-3 activation in a mitochondria independent manner [42, 67]. The prominent caspase-9 activation after 2 h DX treatment was followed by remarkable caspase-3 activation after 3 h DX treatment. The number of DP cells containing activated caspase-9 was almost doubled after 2 h and the number of cleaved caspase-8 containing DP cells increased significantly but to a lesser extent than caspase-9. This observation suggests that the activation of caspase-9 may be prior to caspase-8 activation and strengthen the importance of the mitochondrial apoptotic pathway in DX-induced apoptosis of DP thymocytes.

Our results are supported by the work of other research groups. Several knock-out models have been generated already, where one or more members of the Bcl-2 family or caspases were inactivated and thus, the deficiency of these proteins can be studied effectively. These models have provided an important insight into the different apoptotic pathways. For example, caspase-9^−/−^ KO thymocytes have been found to be resistant to DX-induced apoptosis, but remained sensitive to apoptosis induced by TNF-α, α-CD95 [40]. Apaf^−/−^ KO thymocytes have shown only partial resistance to DX-induced apoptosis and impaired procaspase-8 processing, but were sensitive to apoptosis induced by Fas ligation [41]. GC-induced thymocyte apoptosis has been unaffected in Bid-deficient mice suggesting the dispensable role of the extrinsic apoptotic pathway in GC mediated cell death [68]. On the other hand, using small peptide inhibitors of caspases have shown the importance of caspase-3 and -8 in GC-induced thymocyte apoptosis [42, 43], but the specificity of these inhibitory molecules might be unclear [69–71]. Some results have suggested the primary role of caspase-9 in GC-induced apoptosis [40, 41]. However, others have not supported these findings [42, 72]. The activation of caspase-8 could also be the result of the activation of caspase-9 either through the release of cathepsin B from lysosomes leading to caspase-8 activation [73] or through the activation of caspase-3 and -6, which then cleaves caspase-8 [74]. But the activation of caspase-8 can be the result of the induction of other apoptotic pathways activated by GCs including; ceramide and sphingosine production, Cyclin-dependent kinase 2 activation, or as already mentioned above, the lysosomal release of cathepsin B [42, 72, 73, 75].

**Fig. 6:**
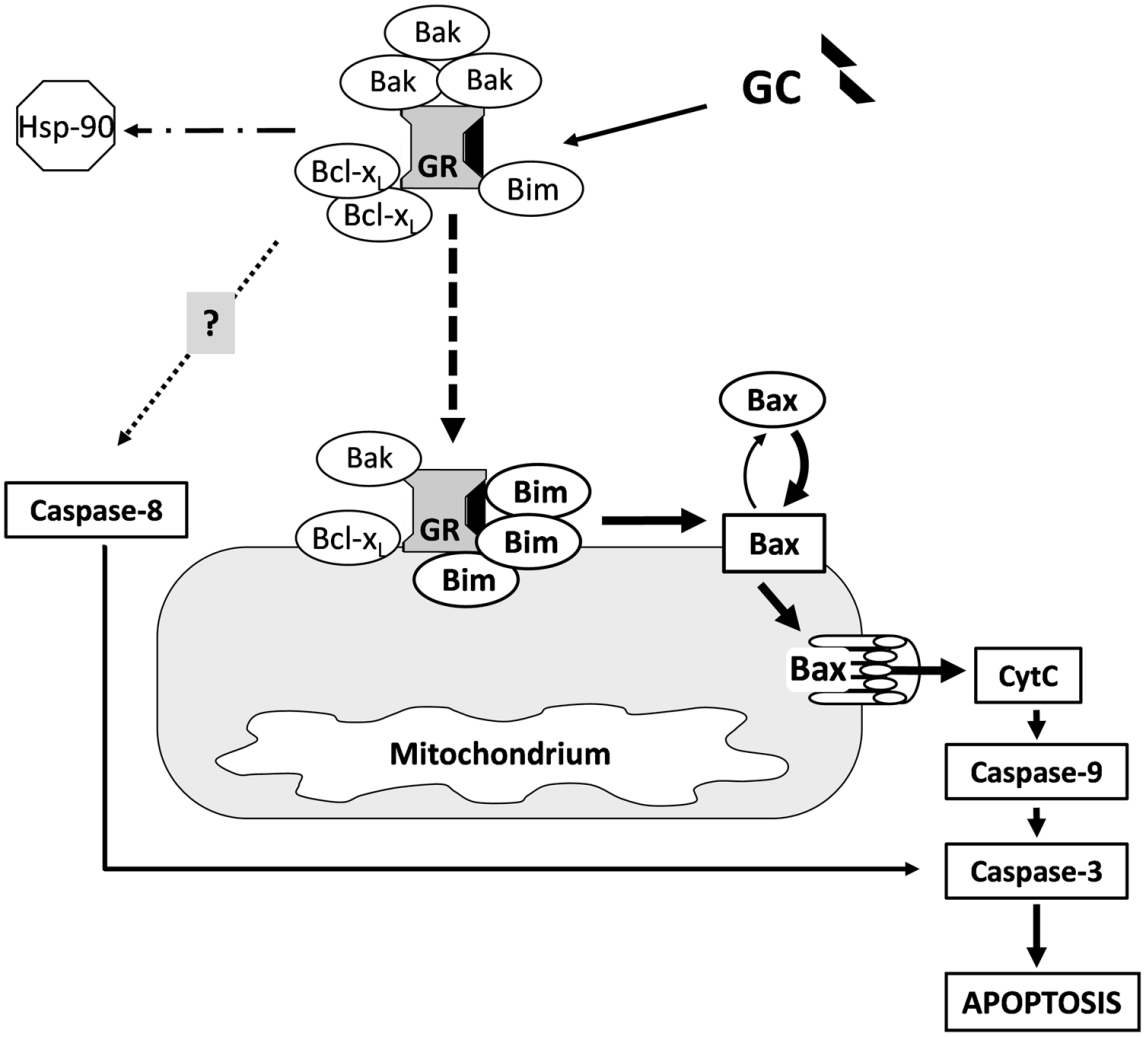
Hypothetical model of the GC-induced apoptosis of thymocytes via the regulation of the mitochondrial apoptotic pathway by members of Bcl-2 protein family. Upon high dose GC treatment the GR translocates to the mitochondria (*dashed arrow*) where its interaction increases with Bcl-2 family proteins, especially with Bim. Then Bax is presumably activated by Bim, leading to permeability pore formation in the mitochondrial outer membrane, and the leakage of Cytochrome *C* into the cytoplasm, which triggers the caspase-cascade. The accumulation of Bax in the mitochondrial outer membrane is most likely further enhanced by the increased cytoplasmic association of the liganded GR and the Bcl-x_L_ which suspends the latter’s inhibitory effect on the mitochondrial pore formation by Bax. The role of the GR-Bak association remains to be elucidated. Caspase-8 activation (extrinsic pathway) may be the result of the interaction between the GR and other apoptotic pathways (*dotted arrow*)

In conclusion, our results demonstate the complexity of early steps of the DX-induced mitochondrial apoptotic pathway in GC sensitive, DP thymocytes (Fig. 6). In the absence of its ligand some association could be observed between the GR and members of the Bcl-2 family (Bak, Bim, Bcl-x_L_) proteins. There is a constant turnover of the pro-apoptotic Bax between the mitochondrial outer membrane and the cytoplasm. When no apoptotic stimuli are present Bcl-x_L_ retrotranslocates Bax from the mitochondrial outer membrane, thus the majority of Bax is located in the cytoplasm in an inactive conformation [62]. Upon high dose GC treatment the liganded GR changes the equilibrium between the Bcl-2 family proteins, in such a way, which promotes apoptosis. GR translocates to the mitochondria where its interaction increases especially with Bim. Bim presumably activates Bax leading to the accumulation and permeability pore formation of Bax in the mitochondrial outer membrane, causing the decrease of the mitochondrial membrane potential [6], the release of Cytochrome *C* and the activation of caspase-9 (Fig. 6). The pore formation of Bax in the mitochondrial outer membrane might be supported by the increased cytoplasmic association of the activated GR with Bcl-x_L_,which interferes with the latter’s inhibitory effect on the mitochondrial pore formation by Bax. The role of the GR-Bak association needs further investigations. Caspase-8 activation (extrinsic pathway) may be the result of the interaction of GR and other apoptotic pathways [42, 72, 73, 75]. Taken together, our results emphasize the importance of the mitochondrial apoptotic pathway and the non-genomic effects in GC-induced thymocyte apoptosis.
